# Population genomics of Australian indigenous *Mesorhizobium* reveals diverse nonsymbiotic genospecies capable of nitrogen-fixing symbioses following horizontal gene transfer

**DOI:** 10.1099/mgen.0.000918

**Published:** 2023-01-05

**Authors:** Elena Colombi, Yvette Hill, Rose Lines, John T. Sullivan, MacLean G. Kohlmeier, Claus T. Christophersen, Clive W. Ronson, Jason J. Terpolilli, Joshua P. Ramsay

**Affiliations:** ^1^​ Curtin Medical School, Curtin University, Bentley, Western Australia 6102, Australia; ^2^​ Curtin Health Innovation Research Institute, Curtin University, Bentley, Western Australia 6102, Australia; ^3^​ Legume Rhizobium Sciences, Food Futures Institute, Murdoch University, 90 South St, Murdoch, Western Australia 6150, Australia; ^4^​ Trace and Environmental DNA (TrEnD) Laboratory, School of Molecular and Life Sciences, Curtin University, Kent Street, Bentley, Western Australia 6102, Australia; ^5^​ Department of Microbiology and Immunology, University of Otago, Dunedin, New Zealand; ^6^​ School of Medical & Health Sciences, Edith Cowan University, Joondalup, Western Australia, Australia; ^7^​ Centre for Integrative Metabolomics and Computational Biology, Edith Cowan University, Joondalup, Western Australia, Australia; ^†^​ Present address: School of Agriculture and Food, Faculty of Veterinary and Agricultural Sciences, The University of Melbourne, Victoria 3010, Australia

**Keywords:** symbiosis, conjugation, symbiosis island, evolution, horizontal gene transfer, ICE, integrative and conjugative elements, *Mesorhizobium*, nitrogen fixation, plant-microbe interactions, rhizosphere, soil bacteria

## Abstract

Mesorhizobia are soil bacteria that establish nitrogen-fixing symbioses with various legumes. Novel symbiotic mesorhizobia frequently evolve following horizontal transfer of symbiosis-gene-carrying integrative and conjugative elements (ICESyms) to indigenous mesorhizobia in soils. Evolved symbionts exhibit a wide range in symbiotic effectiveness, with some fixing nitrogen poorly or not at all. Little is known about the genetic diversity and symbiotic potential of indigenous soil mesorhizobia prior to ICESym acquisition. Here we sequenced genomes of 144 *Mesorhizobium* spp. strains cultured directly from cultivated and uncultivated Australian soils. Of these, 126 lacked symbiosis genes. The only isolated symbiotic strains were either exotic strains used previously as legume inoculants, or indigenous mesorhizobia that had acquired exotic ICESyms. No native symbiotic strains were identified. Indigenous nonsymbiotic strains formed 22 genospecies with phylogenomic diversity overlapping the diversity of internationally isolated symbiotic *Mesorhizobium* spp. The genomes of indigenous mesorhizobia exhibited no evidence of prior involvement in nitrogen-fixing symbiosis, yet their core genomes were similar to symbiotic strains and they generally lacked genes for synthesis of biotin, nicotinate and thiamine. Genomes of nonsymbiotic mesorhizobia harboured similar mobile elements to those of symbiotic mesorhizobia, including ICESym-like elements carrying aforementioned vitamin-synthesis genes but lacking symbiosis genes. Diverse indigenous isolates receiving ICESyms through horizontal gene transfer formed effective symbioses with *Lotus* and *Biserrula* legumes, indicating most nonsymbiotic mesorhizobia have an innate capacity for nitrogen-fixing symbiosis following ICESym acquisition. Non-fixing ICESym-harbouring strains were isolated sporadically within species alongside effective symbionts, indicating chromosomal lineage does not predict symbiotic potential. Our observations suggest previously observed genomic diversity amongst symbiotic *Mesorhizobium* spp. represents a fraction of the extant diversity of nonsymbiotic strains. The overlapping phylogeny of symbiotic and nonsymbiotic clades suggests major clades of *Mesorhizobium* diverged prior to introduction of symbiosis genes and therefore chromosomal genes involved in symbiosis have evolved largely independent of nitrogen-fixing symbiosis.

## Data Summary

All genome assemblies and sequence reads are available in NCBI BioProject PRJNA549135. Amplicon sequencing reads from soil DNA are available in PRJNA841043.

**Impact Statement**

Rhizobia are soil bacteria that can establish nitrogen-fixing symbioses with legumes. In rhizobia of the genus *Mesorhizobium *, genes for symbiosis are often located on mobile integrative and conjugative elements (ICESyms) that horizontally transfer to indigenous mesorhizobia in soils. However, symbiosis gene transfer does not always result in an effective nitrogen-fixing symbiosis, suggesting the genetic background impacts symbiotic performance. Since mesorhizobia are generally isolated from plant root nodules, very little is known about the diversity of indigenous soil *Mesorhizobium* prior to their acquisition of symbiosis genes. In this work we isolated 126 nonsymbiotic *Mesorhizobium* spp. from Australian soils and sequenced their genomes. Nonsymbiotic *Mesorhizobium* exhibited similar core and pangenome features to symbiotic isolates and overlapping phylogenetic diversity. Transfer of ICESyms to diverse nonsymbiotic *Mesorhizobium* spp. produced symbiotic strains with varying nitrogen-fixing efficiency, including some that were ineffective symbionts. Efficacy of symbiosis was not correlated with the lineage of the *Mesorhizobium* host chromosome, suggesting sporadic genetic incompatibilities in recipient genomes augment symbiotic effectiveness. These findings broaden our appreciation of *Mesorhizobium* diversity and reveal Australian soil *Mesorhizobium* populations are dominated by nonsymbiotic isolates that overlap the phylogenetic diversity of symbiotic isolates isolated internationally.

## Introduction

Rhizobia are a group of phylogenetically diverse soil bacteria capable of forming symbiosis with legumes through the formation of root nodules. Within root nodules, rhizobia fix atmospheric nitrogen (N_2_) to produce NH_3_, which is secreted to the plant and assimilated. In return, the host-plant supplies carbon, nutrients and a niche for the microsymbiont. Currently, at least 14 genera within the *Alphaproteobacteria* and *Betaproteobacteria *, are known to establish a legume symbiosis [1]. *Mesorhizobium* strains can establish nitrogen-fixing symbioses with legume species from temperate, tropical, sub-tropical and arctic areas [2]. These include cultivated legumes such as *Lotus* spp., *Biserrula pelecinus* [3, 4] and *Cicer* spp., [5] and a variety of uncultivated legumes [6–10] including *Robinia* [11], *Carmichaelia* [7], *Acacia* and *Sesbania* [12].

Bacterial genes that contribute to rhizobia-legume symbioses include nodulation (*nod*, *noe* and *nol*) and nitrogen fixation (*nif* and *fix*) genes. In *Mesorhizobium* spp., symbiosis genes are generally present on integrative and conjugative elements (ICEs) [13], which are chromosomally integrating mobile genetic elements (MGEs) that can excise from the chromosome and transfer using ICE-encoded conjugation machinery [14]. The first-described 'symbiosis ICE' (ICESym), ICE*Ml*Sym^R7A^, is a 502 kb ICE enabling nitrogen-fixing symbiosis with *Lotus corniculatus*. Transfer of ICE*Ml*Sym^R7A^ converts nonsymbiotic *Mesorhizobium* spp. into nitrogen-fixing symbionts of *L. corniculatus* [15]. Related ICESyms convert indigenous Australian *Mesorhizobium* spp. into microsymbionts of *B. pelecinus* [3, 4] and *Cicer arietinum* (chickpea) [16]. ICESyms form a subfamily within a much larger family of ICEs distributed throughout the proteobacteria, and are the largest of all characterized bacterial ICEs at ~350–850 kb [13]. ICESym-encoded site-specific recombinases target conserved chromosomal loci such as tRNA genes for integration. Tripartite ICESyms possess three distinct site-specific recombinases and following initial integration, two further inversions between the ICE and chromosome separate the tripartite ICE genome into three regions (*α*, *β* and *γ*) [17, 18]. Both monopartite and tripartite *Mesorhizobium* ICESyms have likely evolved from a common ancestor and carry related core genes for quorum sensing, conjugation, excision and regulation [13].

In Australia and New Zealand (NZ), where cultivated legumes have been introduced following European colonization [19, 20], soils often lack indigenous compatible microsymbionts [21–23], so inoculation with compatible rhizobia is a common agricultural practice [19, 24]. Several studies reveal rhizobia re-isolated from nodules of inoculated legumes are often genetically distinct to the inoculant strain [23, 25, 26] due to gene transfer from the inoculant to indigenous rhizobia. In some cases, ICESym-transfer to *Mesorhizobium* spp. in Australian soils produce exconjugants that fix nitrogen sub-optimally [27] or not at all [28], so the genetic background of indigenous *Mesorhizobium* spp. impacts the efficacy of legume inoculation. The genetic composition of the Australian indigenous *Mesorhizobium* spp. prior to ICESym acquisition is unknown. However, diverse nonsymbiotic mesorhizobia can be isolated from NZ soils and can acquire ICE*Ml*Sym^R7A^ both *in situ* and *in vitro* [29].

Few studies have focussed directly on populations of nonsymbiotic rhizobia; however, most reveal diverse nonsymbiotic rhizobia coexist with symbiotic rhizobia in soils. Early studies demonstrated nonsymbiotic strains related to *Rhizobium leguminosarum* bv. *phaseoli* were converted into effective legume symbionts following transfer of symbiosis plasmids [30–32]. Diverse nonsymbiotic *Rhizobium* spp. coexist in rhizospheres with symbiotic rhizobia but symbiosis-plasmid transfer seems restricted between closely related species [33]. The diversity of *Bradyrhizobium* species in North American soils suggests populations are dominated by a few abundant lineages comprising both symbiotic and nonsymbiotic bacteria [34, 35]. In *Bradyrhizobium* spp., symbiosis genes are present on chromosomally integrated elements [36] and while many appear to have been independently acquired, many may have been subsequently lost from the same lineages [34]. Symbiotic and nonsymbiotic *Ensifer* (syn. *Sinorhizobium *) spp. appear to form monophyletic symbiotic (* Sinorhizobium *) and nonsymbiotic (* Ensifer *) clades, with few exceptions, but these are now recognized as two different genera [37]. Nonsymbiotic isolates are also present in the *Sinorhizobium* genus and symbiosis plasmids have been acquired in several independent events [38]. Together, these observations highlight the complex interplay between MGEs carrying symbiosis genes and their bacterial hosts.

Members of the genus *Mesorhizobium* lacking genes for nitrogen-fixing legume symbiosis have been isolated from nodules [39], sea water [40], activated sludge used in wastewater treatment [41], photosynthetic amoeba [42], *Arabidopsis* root microbiota [43] and even human blood [44]. Australian soils appear devoid of *Mesorhizobium* capable of nodulating introduced legumes but indigenous *Mesorhizobium* spp. acquiring ICESyms from inoculant strains readily evolve. It is unclear if most indigenous *Mesorhizobium* can become nitrogen-fixing symbionts upon ICESym acquisition or if these exconjugants represent a subset of the population. In this work, we isolated 144 *Mesorhizobium* directly from cultivated and uncultivated Australian soils. Of these, 126 strains were nonsymbiotic *Mesorhizobium* spp. (NS-meso). Genome comparisons suggested NS-meso genomes are similar in composition and diversity to symbiotic isolates. We propose the NS-meso isolated in this study reflect the archetypal diversity present in the genus prior to the evolution of nitrogen-fixing symbiosis and that most NS-meso have an ‘innate’ capacity to evolve into nitrogen-fixing symbionts following ICESym acquisition.

## Methods

Detailed methods are provided in the supplementary material. Software commands used are available at https://github.com/EC-Rufina/NS-Meso and https://github.com/EC-Rufina/MesICE.

### Isolation of *Mesorhizobium* from soil and sequencing

Four-gram soil samples (Table S1 available in the online version of this paper) were washed with sterile water, and dilutions in 10 mM MgCl_2_ were plated on G/RDM [45, 46] containing cycloheximide (100 µg ml^−1^), quintozene (5 µg ml^−1^), nystatin (50 µg ml^−1^), fosfomycin (50 µg ml^−1^), nicotinate (1 µg ml^−1^), biotin (20 ng ml^−1^) and thiamine (1 µg ml^−1^), and incubated at 28 °C for 10 days. Single colonies were PCR-screened with *Mesorhizobium *-specific 16S-rRNA primers (Table S2). Genome extractions for Oxford Nanopore Technologies MinION and PacBio RSII sequencing were performed as previously described [47]. For assembly of complete genomes with MiniION or PacBio reads, long-reads alone were first assembled with Flye 2.7.1 [48] and then assemblies were polished with Illumina reads as previously described [49]. Genome extractions for Illumina NextSeq sequencing were performed with FavorPrep Tissue Genomic DNA Extraction Mini Kit (Favorgen). Illumina adapters were removed with Trimmomatic 0.39 [50], reads were corrected using Lighter (v1.1.1) [51]. Genomes sequenced only with Illumina reads were assembled with SPAdes 3.5.0 [52].

### Genome analyses

Phylogenies were constructed as described previously [13], except PRANK [53] was used for alignments. Trees were plotted with ggtree [54]. Pangenomes were evaluated with Proteinortho [55]. NodABC and NifHDK searches were performed as previously described [38]. *Mesorhizobium* and *Rhizobium leguminosarum* Rlv3841 genes involved in symbiosis were initially searched for in *M. japonicum* R7A with blastp, and Proteinortho [55] was used to identify orthologs of R7A proteins in other *Mesorhizobium* genomes. Identification of MGEs was performed as previously described [13]. Each MGE was queried against other genomes with blastn and considered present in other strains if >80 % pairwise nucleotide identity and >80 % coverage were shared.

### Microbiome analysis

Soil DNA was isolated using DNeasy PowerSoil Kit (QIAGEN). Barcoding qPCRs with 16S V4 and *atpD* primers (Table S3), and sequencing were carried out as in [56]. Reads were processed using mothur 1.32.1 software as per the MiSeq standard operating procedure [57]. PCR errors were accounted for by removing any sequences with ambiguous bases. For the 16S amplicon sequencing, reads longer than 275 bp were removed, for the *atpD* amplicon sequencing reads longer than 300 bp and reads shorter than 294 bp were discarded. Potential chimaeras were identified and removed using the mothur chimaera.vsearch command with default parameters.

### ICE*Ml*Sym^R7A^ transfer experiments

ICE*Ml*Sym^R7A^ was conjugated from strain R7A* as in [49]. Briefly, donor and recipient strains were mixed and spotted on a TY agar plate and incubated at 28 °C for 24 h. Exconjugants were selected on G/RDM containing tetracycline (50 µg ml^−1^) or neomycin (200 µg ml^−1^), without supplemented nicotinate, biotin or thiamine.

### Legume symbioses

Pots containing sterile sand mix or soil collected from Badgingarra were sown with germinated *B. pelecinus* seeds as previously described [58]. Pots were sown with four germinated seeds and four replicates for each treatment. Plants were grown for 69 days. Nodule collection and treatments were conducted as previously described [59, 60]. Shoots were dried at 60 °C and desiccated prior to weighing. *Lotus japonicus* ecotype Gifu [61] was used to test the symbiotic capacity of R7A and exconjugants as previously described [62].

## Results

### Diverse nonsymbiotic mesorhizobia can be isolated from Australian soils

**Fig. 1.**
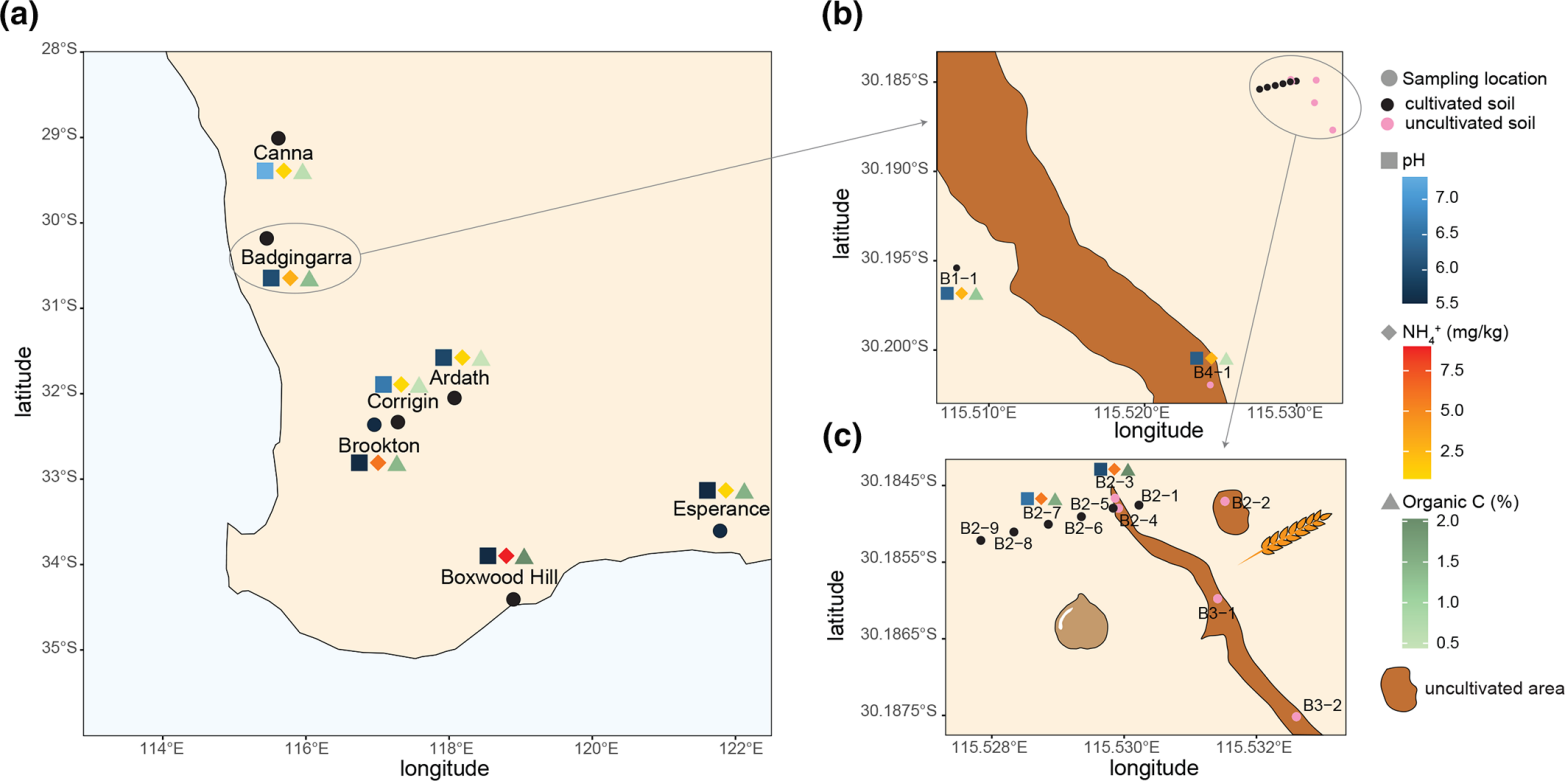
Map of soil sites. (a) Sampling sites across western Australia. (b),(c) Location of the sampling sites in Badgingarra. Soils were analysed for pH, and content of NH_4_^+^ and organic C, results are indicated by squares, diamonds or triangles coloured as indicated in the key on the right. The chickpea seed and the ear of wheat cartoons in (c) indicate that the paddocks were cultivated with these crops at the time of sampling.

Soil was collected from a 0–20 cm profile in areas of cultivated and uncultivated land in western Australia (Figure 1, Table S1). Thirteen soil samples were taken from a farm in Badgingarra (sites B1-1 to B4-1), including paddocks subjected to rotational cropping of grains and legumes, and neighbouring uncultivated areas containing native *Eucalyptus* spp., *Daviesia* spp., and various weed grass species (Fig. 1b, c). An additional six samples across southern-western Australia were taken from cultivated farmland with diverse soil characteristics (Fig. 1a).

To isolate *Mesorhizobium* from soil and reduce contaminating organisms, a modified semi-selective glucose rhizobium defined medium (G/RDM [45, 46]) was developed (see Methods for complete medium recipe). Antimicrobial susceptibility screening of *Mesorhizobium* strains in our collection revealed all were highly resistant to fosfomycin. We suspected this was partly due to strains carrying *fosX*. Deletion of *fosX* in *M. japonicum* R7A only reduced the fosfomycin resistance from >1 g ml^−1^ to a minimum inhibitory concentration of ~300 mg ml^−1^. Deletion of *fosX* in *M. ciceri* CC1192 reduced fosfomycin resistance from >900 mg ml^−1^ to a minimum inhibitory concentration of ~100 mg ml^−1^. This suggests that *Mesorhizobium* species are resistant to fosfomycin through various molecular mechanisms [45, 46]. A final concentration of 50 mg ml^−1^ fosfomycin was used in the isolation medium along with vitamins nicotinate, biotin and thiamine and antifungals cycloheximide, quintozene and nystatin.

Soil washes were plated on solid semi-selective medium described above and incubated for 10 days. Approximately 900 individual colonies with mucoid and convex morphology were PCR-screened using *Mesorhizobium *-specific primers (Table S2), and 150 isolates were selected for short-read genome sequencing. 16S rRNA-gene sequences confirmed 144 isolates were *Mesorhizobium* spp. (Table S3). Of these, 126 were nonsymbiotic *Mesorhizobium* (NS-meso) and only 18 isolates carried nodulation (*nodABC*) and nitrogenase (*nifHDK*) genes. Of these, four strains shared 99.9 % average nucleotide identity (ANI) across their entire genome with the commercial chickpea inoculant *M. ciceri* CC1192. The remaining 14 isolates were not identified as re-isolated commercial inocula, however, blastn searches using ICESyms (as previously described [13]) revealed they were NS-mesos that had acquired the ICESym of CC1192 (one isolate) or the ICESym of the *Biserrula* inoculant strain *M. ciceri* bv. *biserrulae* WSM1497 (Table S3).

**Fig. 2.**
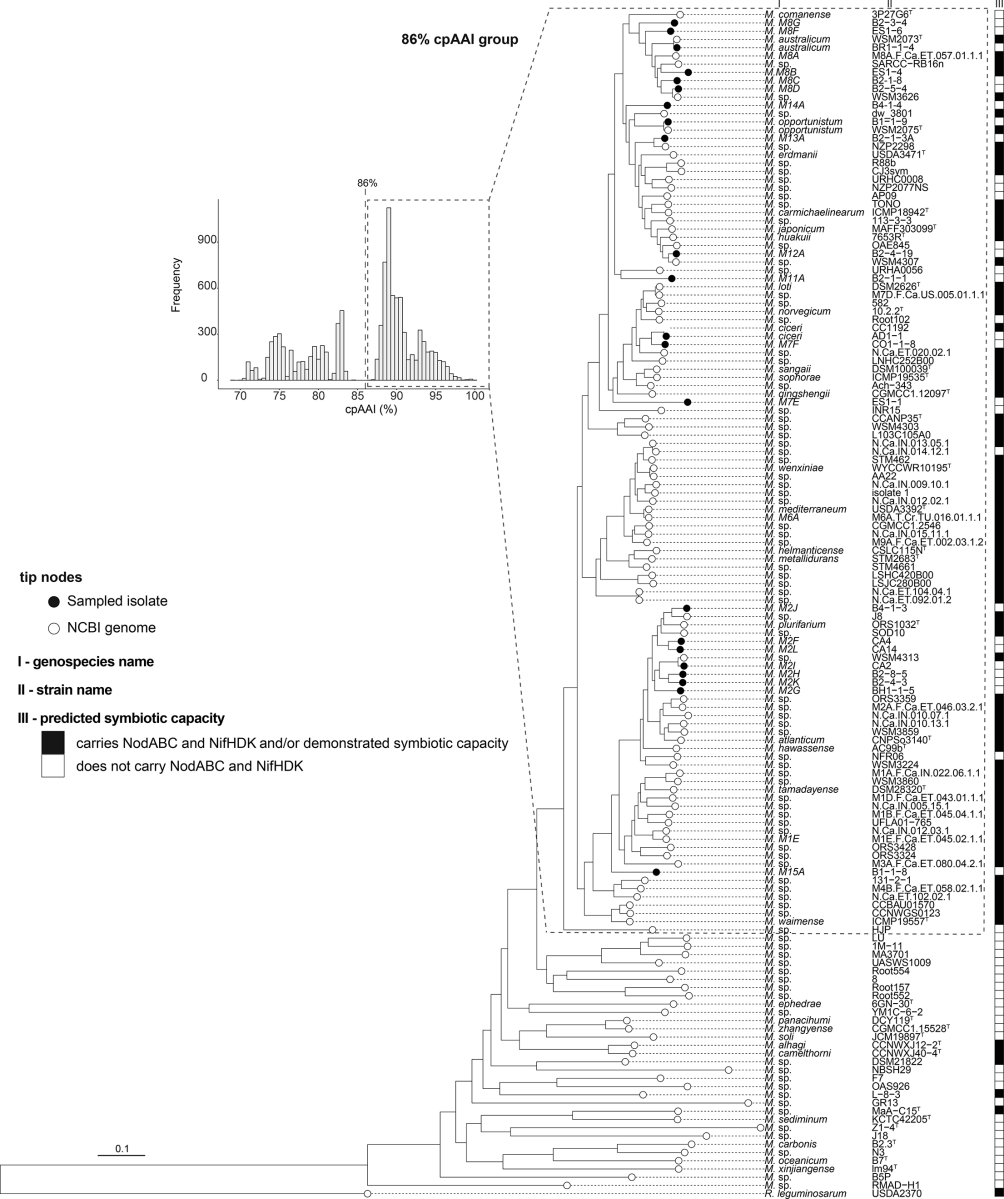
Overview of the *Mesorhizobium* genus. Maximum-likelihood tree constructed on 436 single-copy core genes with RAxML. The tree was rooted with the type-strain *Rhizobium leguminosarum* biovar *viciae* USDA2370 (MRDL00000000). Strains were predicted to be symbiotic if they harboured NodABC and NifHDK genes and/or if they had published symbiotic capacity, and were assumed nonsymbiotic if they lacked these genes or demonstrated symbiotic capacity. Unfilled circles at tip nodes indicate NCBI-downloaded reference genomes (type strains are indicated by ^T^), and black-filled circles at tip nodes indicate *Mesorhizobium* isolated in this study. Scale bar indicates substitutions per site. The histogram in the upper-left displays the distribution of core-proteome amino-acid identity (cpAAI) (bin width 0.5 %) calculated from the same 436 core genes used in tree generation, and the dotted line indicates species within the cpAAI>86 %.

Pairwise whole-genome comparisons clustered the NS-meso into 22 genospecies with ANI>95 %. To name novel genospecies, we extended a scheme used by Greenlon *et al.* [5]. Briefly, novel genospecies falling in clades (M-number) were designated an M-number and a new letter (e.g. *M.* sp. M2I), and new clades and genospecies were assigned a new M-number and letter (e.g. *M.* sp. M11A). A maximum-likelihood core-gene phylogeny (Fig. 2) was constructed using a representative from each NS-meso genospecies together with *Mesorhizobium* spp. genospecies representatives deposited in NCBI (Table S4 and Table S5). This tree segregated most symbiotic *Mesorhizobium* spp. (which included 22 type strains, all symbiotic) from previously isolated nonsymbiotic *Mesorhizobium* spp. (which included 11 nonsymbiotic type strains and two symbiotic type strains). The ‘symbiotic cluster’ shared a pairwise core-proteome average amino acid identity (cpAAI) of >85–86 %, a measure previously deemed suitable to delineate *Rhizobiaceae* genera and other genera [37, 63, 64] (Fig. 2). The distribution of cpAAI scores revealed a clear demarcation between genospecies sharing >87 % cpAAI and others sharing <84 % cpAAI, suggesting those with less than ~86 % cpAAI could be considered distinct genera. All NS-meso isolated here grouped within the ‘symbiotic' cpAAI>86 % cluster. NS-meso were dispersed throughout the cpAAI>86 % cluster, and of the 22 genospecies isolated, only seven genospecies had been previously sequenced (Table S6).

**Fig. 3.**
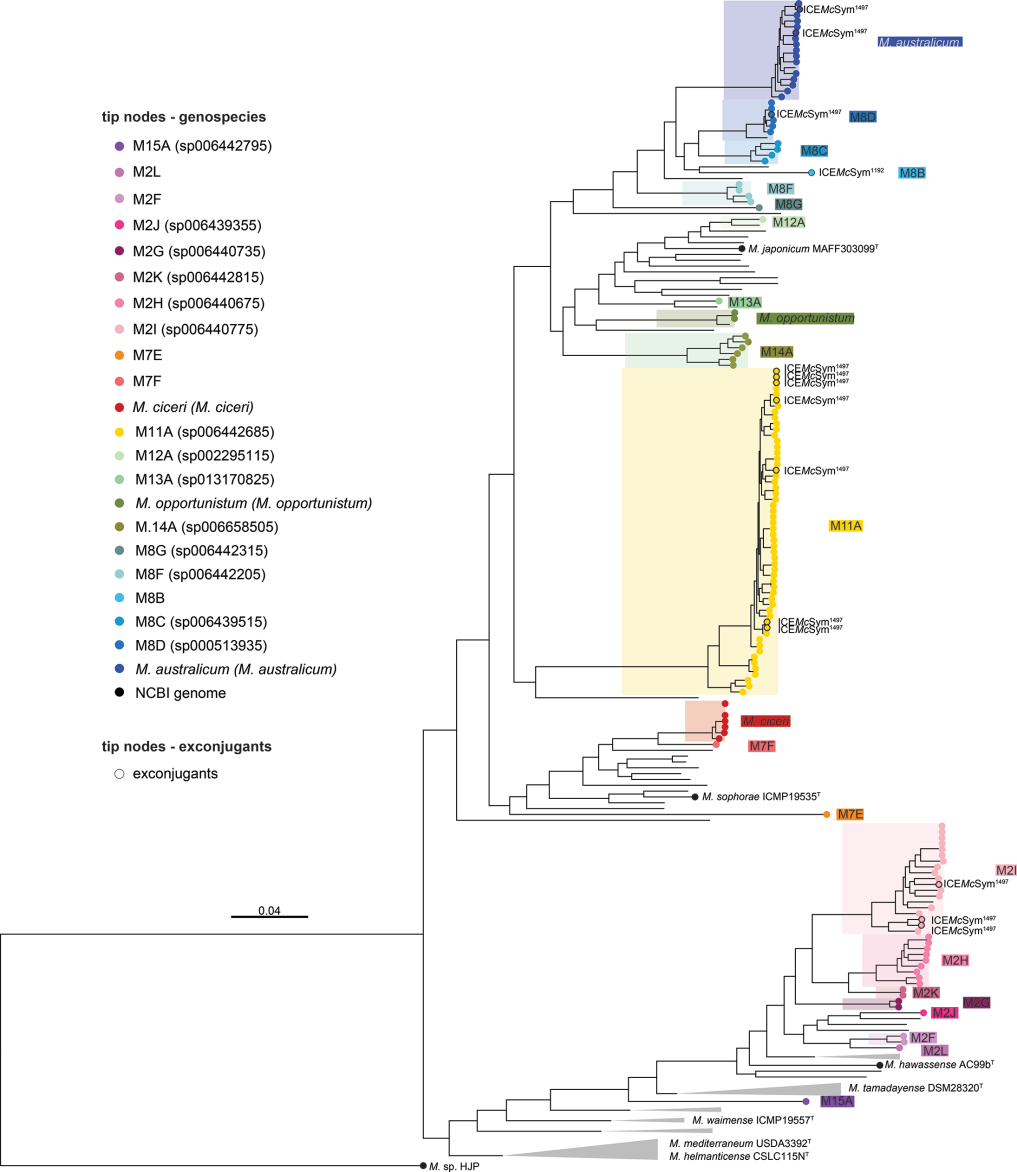
Core-gene phylogeny of *Mesorhizobium* isolated from soil during this study. In total, 1,067 single-copy core-genes were used to build a maximum-likelihood tree using RAxML. The tree was rooted with the *M.* sp. HJP. Coloured tip nodes indicate the strains were isolated in this study, with colour of the tip nodes representing the species the strain belongs to. The genospecies name attributed by the Genome Taxonomy Database is reported in brackets. Coloured tip nodes with a black border indicate that the strain carries an ICESym of a commercial inoculum, the name of the ICESym is indicated adjacent to the tip node. Other genomes were downloaded from NCBI, grey triangles indicate a collapsed clade and black tip nodes highlight selected type strains. A complete tree with all strain labels is presented in Fig. S1. Scale bar indicates substitutions per site.

To further evaluate relationships between NS-meso and symbiotic strains, a maximum-likelihood core-genes phylogeny was constructed with NS-meso together with previously sequenced *Mesorhizobium* grouped within the cpAAI>86 % threshold (Figs 3 and S1). The NS-meso included documented recipients of the tripartite ICESym ICE*Mc*Sym^1271^, *M. opportunistum* and *M. australicum* [65], and recipients of ICE*Mc*Sym^1192^ [16], genospecies M2I and M12A. Other previously isolated genospecies included those from legume nodules in the Mediterranean basin (* M. ciceri *), NZ, USA, Canada and Japan (genospecies M13A), and in South Africa (genospecies M8D) (Table S6). Of the 15 novel genospecies, M2F to M2L clustered with members of the previously defined ‘clade M2’ isolated from *Cicer* spp. in Ethiopia, India and Morocco [5], and with symbiotic strains isolated from Brazil (* M. atlanticum* CNPSo3140), Japan (*M*. sp. J8), Eritrea (*M.* sp. WSM3859), South Africa (*M.* sp. WSM3224) and Senegal (* M. plurifarium* ORS1032). Seven novel genospecies M8B-M8G, and *M. australicum *, clustered with genospecies M8A, previously identified in India [5]. Several genospecies isolated in this study have also been assigned species names by Genome Taxonomy Database (GTDB) and these are indicated in Fig. 3. The most common NS-meso genospecies was M11A, isolated 56 times across five of the seven locations sampled. M11A was particularly abundant in the Badgingarra soil, representing 46.3 % of strains isolated. The 14 ICESym exconjugants isolated from soil here were also distributed throughout the NS-meso phylogeny (Fig. 3).

A rarefaction curve generated from genospecies isolated here did not plateau (Fig. S2), and abundance-based coverage estimation suggested eight genospecies remain undiscovered [66]. To further estimate diversity of *Mesorhizobium* spp. in soil, amplicon sequencing of DNA from each of the Badgingarra soil samples was carried out using primers for the 16S rRNA gene and a 330 bp region of the *atpD* gene conserved across the genus (Table S2). Approximately 0.001 % of 16S rRNA amplicons (Table S7) and ~2.45 % of *atpD* amplicons were classified as *Mesorhizobium* using mothur [67], which estimated 232 operational taxonomic units (OTU) of unclassified *Mesorhizobium* (Table S8). A tree constructed from *atpD* amplicon sequences together with *atpD* extracted from genome sequences (Fig. S3) placed the amplicon-derived *atpD* sequences throughout the tree, suggesting the additional sequences reflected unsampled diversity within the identified clades isolated here. The M11A OTU was present in 10 of the 13 sites, consistent with its frequent isolation. Overall, these observations suggest that while culture-based sampling underestimated species-level diversity, the phylogenetic distribution of isolated samples correlated well with amplicon sequences, suggesting the culture-based sampling strategy was relatively unbiased.

### Soil and nodule-isolated ICE*Mc*Sym^1497^ exconjugants are capable of nitrogen-fixing symbioses with *Biserrula*

**Fig. 4.**
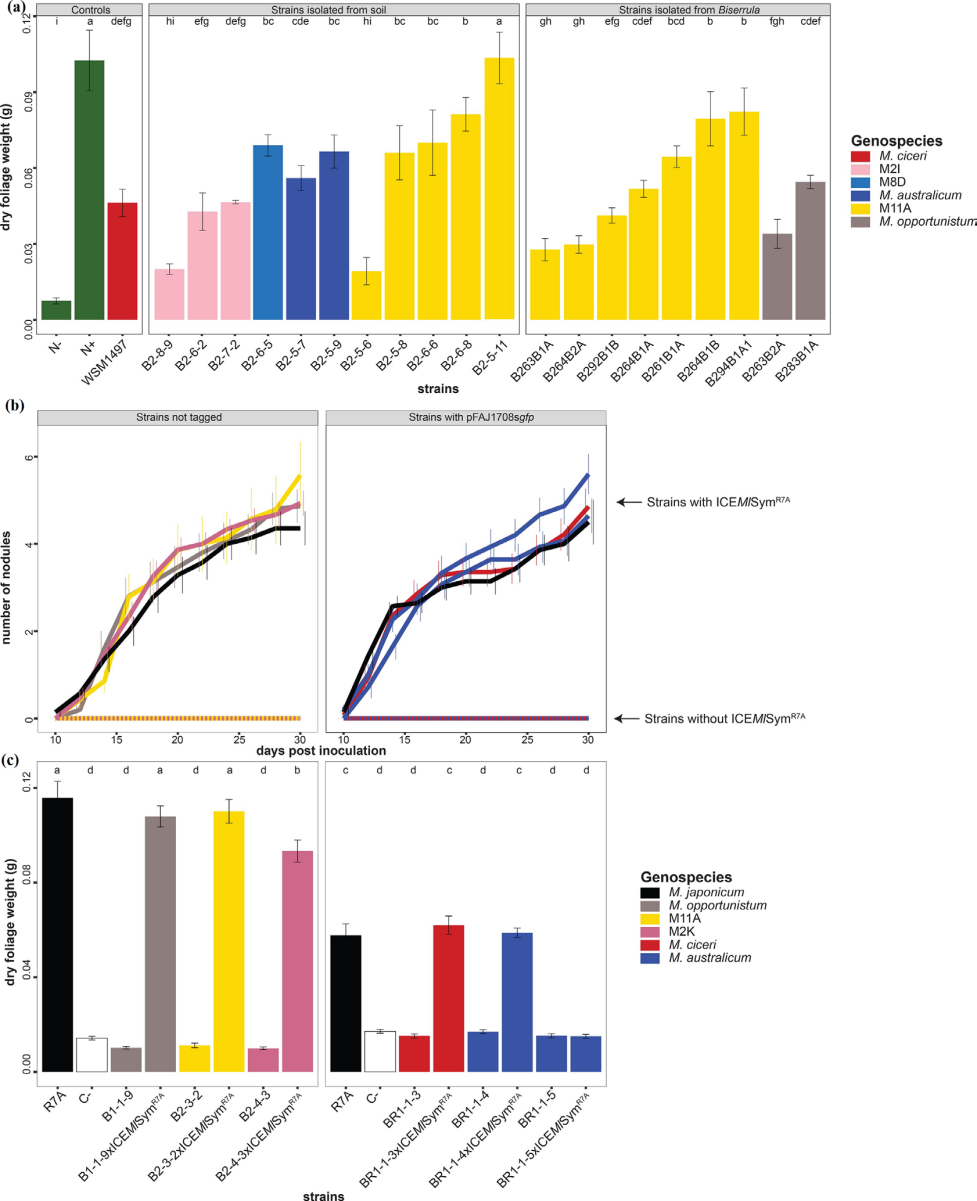
Legume symbioses with NS-meso exconjugants carrying ICESyms. (a) *B. pelecinus* cv. Casbah dry foliage weight at 69 days post-inoculation. Uninoculated (N-) and N-fed (N+) (supplied as KNO_3_) plants were included as negative and positive controls, respectively. Treatments are shown with standard errors of the means, and the treatments that share a letter are not significantly different according to the least significant difference test (*P* ≤ 0.05). (b) Number of nodules developed by the *L. japonicus* Gifu plants grown in test tubes. (c) *L. japonicus* Gifu foliage weight at 30 days post-inoculation. The experiment was performed with 15 replicates and repeated three times. Treatments are shown with standard errors of the means, and treatments that share a letter are not significantly different according to the least significant difference test (*P* ≤ 0.01).

Previous examination of Australian indigenous *Mesorhizobium* spp. carrying ICE*Mc*Sym^1192^ transferred from chickpea inoculant CC1192, or ICE*Mc*Sym^1271^ from the *Biserrula* inoculant WSM1271, indicated ICE*Mc*Sym^1192^ exconjugants can be as effective as CC1192 in nitrogen fixation [16, 68] but most ICE*Mc*Sym^1271^ exconjugants perform poorly compared to WSM1271, or do not fix nitrogen at all (Fix^-^) [69]. With our sampling, we isolated 13 indigenous strains that had acquired the tripartite ICE*Mc*Sym^1497^ of *M. ciceri* WSM1497, the present commercial inoculant for *B. pelecinus* in Australia. Seven were of the prevalent M11A lineage, two were *M. australicum *, three were genospecies M2I and one was M8D. We isolated eight further ICE*Mc*Sym^1497^ exconjugants from *B. pelecinus* cv. Casbah plant nodules grown in glasshouse conditions with the Badgingarra soil samples (and additionally sequenced their genomes, Table S9)*.* Of these, seven isolates were genospecies M11A, while one was a strain of *M. opportunistum.* Seventeen soil or nodule-isolated exconjugants were tested on *B. pelecinus* cv. Casbah. All induced nodule formation and increased plant dry weights to varying degrees compared to N- controls. Only one strain of genospecies M11A and one M2I strain induced nodule formation but exhibited a Fix^-^ phenotype (Fig. 4a, Table S10). Thus, these experiments confirmed our previous observations that there is a wide range in symbiotic effectiveness amongst individual exconjugants carrying the same ICESym. These data also clarified that while the *Mesorhizobium* strain genetic background played an important role, the lack of symbiotic effectiveness sporadically observed for individual isolates was not associated with them belonging to a particular genospecies.

During isolation of *Mesorhizobium* from *B. pelecinus* cv. Casbah nodules described above, we isolated an additional *M. opportunistum* strain*,* B283B1A, which carried an ICESym distinct from those previously characterized (Table S9). Further interrogation of the farm-site history revealed it was inoculated in 1996 with WSM1558, a strain isolated in Italy for consideration as a commercial inoculant [70]. The genome of WSM1558 was sequenced to completion using long- and short-read sequencing (CP097252). B283B1A genome contigs were aligned with the completed WSM1558 genome, which revealed it was near-identical (99.99 % coverage, 99.99 % nucleotide identity), suggesting that it was a descendant of WSM1558 introduced 25 years ago. Unlike *M. opportunistum* WSM2075 (harbouring ICE*Mc*Sym^1271^), which forms Fix^-^ *B. pelecinus* nodules [65], *M. opportunistum* B283B1A formed an effective symbiosis with *B. pelecinus* cv. Casbah (Fig. 4a).

### Phylogenetically diverse NS-meso can form effective symbiosis with *Lotus japonicus* following laboratory transfer of ICE*Ml*Sym^R7A^

To further explore the capacity of NS-meso to become legume symbionts, we transferred ICE*Ml*Sym^R7A^ from strain *M. japonicum* R7A* [49] to NS-meso strains in laboratory conjugation experiments. ICE*Ml*Sym^R7A^ was successfully introduced into six NS-meso belonging to genospecies *M. opportunistum *, *M. ciceri *, *M. australicum *, M11A and M2K (Table S11). The ICE*Ml*Sym^R7A^ exconjugants were tested on *L. japonicus* Gifu (Fig. 4b and c). While none of the NS-meso strains lacking ICE*Ml*Sym^R7A^ were able to induce nodule formation, the same strains carrying ICE*Ml*Sym^R7A^ exhibited similar nodulation kinetics to R7A. Exconjugants of B1-1-9, B2-3-2 and BR1-1-3 produced plants with weights not statistically different from R7A. One of the two *M. australicum* exconjugants exhibited a Fix^-^ phenotype (Fig. 4c). As observed in the above experiments with *Biserrula,* these data support the notion that most NS-meso genospecies have an innate capacity to form nitrogen-fixing symbioses following ICESym acquisition and that Fix^-^ exconjugants arise sporadically in several genospecies.

### NS-meso and symbiotic *Mesorhizobium* have similar core genomes

The above experiments suggest most NS-meso have a capacity to form nitrogen-fixing symbioses with legumes following ICESym acquisition. This implies that genes not encoded by ICESyms, but nonetheless essential for symbiosis, are highly conserved on the *Mesorhizobium* chromosome. Pangenome analysis of the NS-meso genomes estimated a pangenome of 30,249 genes and a core genome (defined as genes present in all the strains) of 2,469 protein-coding genes. This is significantly higher than our previous estimate of 1,670 core genes for 41 closed and completed genome sequences of the genus *Mesorhizobium* [13]. However, that analysis included distantly related nonsymbiotic members outside the >86 % cpAAI threshold. For a more direct comparison, the ‘symbiotic’ pangenome was recalculated for 19 completely sequenced nitrogen-fixing symbiotic mesorhizobia within the 86 % cpAAI threshold with their ICESym sequences removed, which produced a core genome estimate of 3,149. Core genomes of NS-meso and 19 symbiotic mesorhizobia overlapped by 74 %, yet 813 genes considered core in symbiotic strains were not core in NS-meso genomes.

To gauge the likelihood that the average NS-meso strain had a genetic capacity to become a legume symbiont following ICESym acquisition, we searched genomes for genes involved in nodulation and nitrogen-fixation in *Mesorhizobium* spp. (Table S12), and for homologues of proteins involved in symbiosis and rhizosphere/host colonization identified from *R. leguminosarum* bv. *viciae* Rlv3841 insertion-sequencing experiments [71] (Table S13). This revealed that, aside from symbiosis-associated genes typically found on ICESyms, genes involved in symbiosis that were defined as core genes in the 19 symbiotic strains were also core genes in NS-meso genomes. Of ~600 symbiosis-associated genes, only 26 were classified as core genes in symbiotic strains but not in NS-meso (Tables S12 and S13). Seventeen of these were absent in symbiotically effective exconjugants identified here (Fig. 4), suggesting they were not essential for symbiosis. The remaining nine core genes absent in NS-meso were homologues of Rlv3841 proteins with putative functions in thiamine-binding, nitrate/sulfonate/bicarbonate transport, haem-export, transcriptional regulation, a maltose O-acetyltransferase, an amido-phosphoribosyl-transferase, a TetR/AcrR-family transcriptional-regulator and a N-acetyltransferase. Overall, these analyses indicated genes likely critical for nitrogen fixation and symbiosis that are not present on ICESyms are generallypresent in the genomes of NS-meso.

### Characterization of the NS-meso mobilome and identification of archetypal members of the ICESym family

We previously characterized the mobilomes of 41 completely sequenced *Mesorhizobium* strains, and revealed that ICEs and integrative and mobilizable elements (IME) are predominant over plasmids and bacteriophages. Here we identified MGEs in NS-meso genomes by searching for conjugative relaxase (MOB) proteins using hidden Markov models and inspecting nearby sequences to identify plasmids, ICEs and IMEs [13]. The genomes of B2-1-1 (M11A), B1-1-8 (M15A), B2-8-5 (M2H), B2-1-8 (M8C) and B4-1-4 (M14A) were completed with long-read PacBio and/or ONT MinION sequencing (Table S3) to facilitate MGE demarcation (Table S14). We identified 298 MOB-encoding genes in the NS-meso genomes (11 strains of five different genospecies did not harbour any MOB gene) (Table S15). Excluding ICESyms of commercial inoculants, 34 ICEs and 35 plasmids were identified. Ninety-four IMEs were related to IME*Ml* ^R88b^-1, a common IME identified in numerous symbiotic strains [13]. Novel IMEs were identified in CO1-1-4, AD1-1-1 and B2-4-6 (Table S15). Overall, 135 contigs with a MOB-encoding gene could not be accurately categorized due to fragmentation of sequence contigs. Each recognized MGE and each contig carrying a MOB gene was compared with all other NS-meso genomes to evaluate their distribution. This revealed individual MGEs were largely restricted to a single genospecies, except for ICESyms and IMEMsp.^CA2^ (Figure S4), which was present in M2I and M2L strains isolated from the same soil sample. IMEMsp.^CA2^ carries putative heat-shock response and circadian oscillator KaiBC genes [72] and a *traACD* mobilisation locus related to that of IME*Ml* ^R88b^-1 [73].

**Fig. 5.**
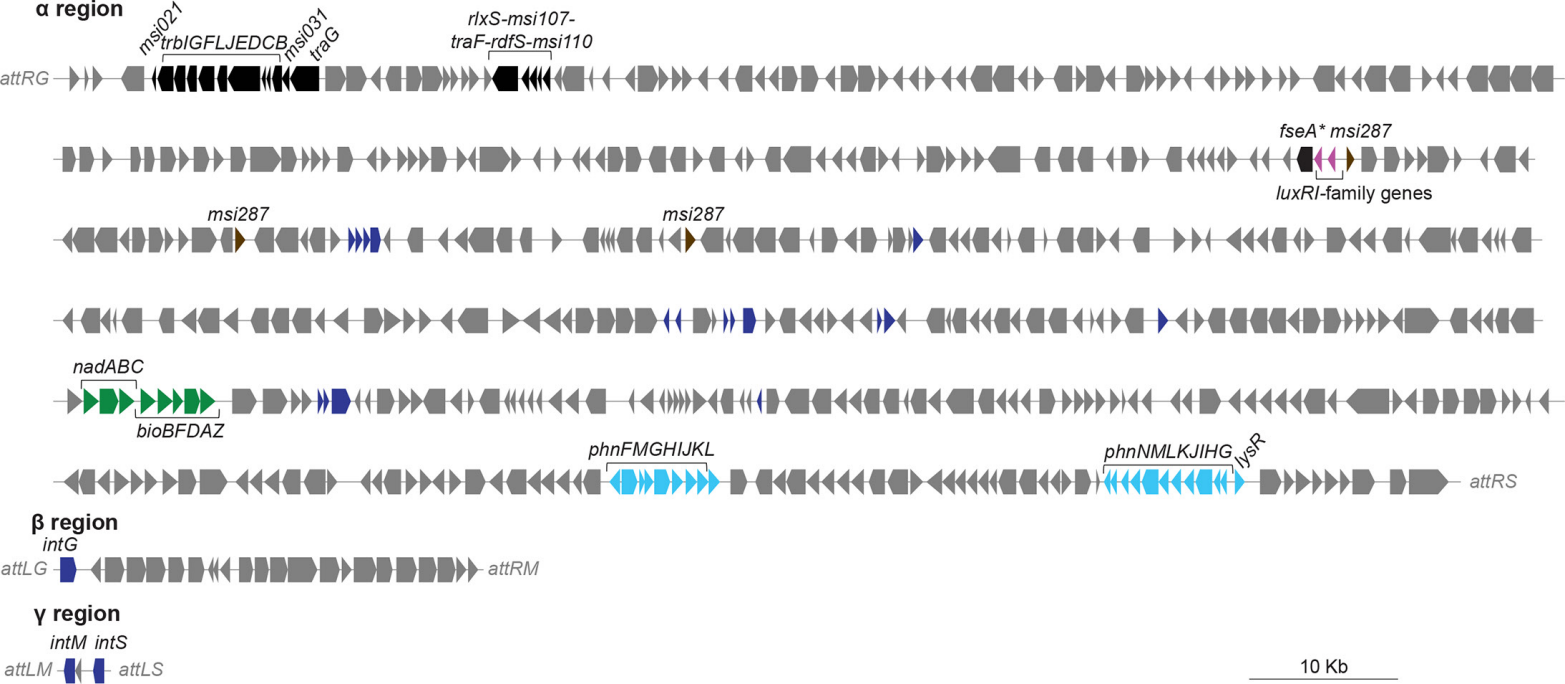
ICE*M*sp^B4-1-4^, a tripartite ICE lacking symbiosis genes. Gene annotations are colour coded as follows: black, ICE transfer genes; purple, quorum-sensing genes; brown, *msi287*; green, vitamin biosynthesis; blue, mobile genes such as transposases, integrases, and recombinases; azure, genes likely involved in the broad-specificity catabolism of phosphonates. An asterisk (*) indicates frameshifting required for gene to be translated.

All ICEs identified here and identified previously in *Mesorhizobium* strains, carry related genes for conjugation and regulation, typified by the presence of unique genes flanking the type IV secretion system (*trb-*gene) cluster (*msi031* and *msi021*) and conserved genes for regulation and DNA transfer, *fseA*, *rdfS* and *rlxS* [13]. We identified 76 *msi031-trb-msi021-*gene clusters (Table S16). Trees were constructed from alignments of the *msi031-trb-msi021-*gene clusters together with clusters previously identified on ICEs and plasmids in *Mesorhizobium* [13]. The various NS-meso *msi031-trb-msi021-*gene clusters were interspersed throughout the tree amongst previously identified *msi031-trb-msi021* clusters (Fig. S5). Interestingly, three of the NS-meso ICE *msi031-trb-msi021-*gene clusters grouped with the ICESym clusters (Figs 5A and S5), and like all other ICESyms (but not other ICEs/plasmids in *Mesorhizobium *), lacked the *trbK* gene. This indicated these ICEs shared a recent common ancestor with the ICESyms despite them lacking symbiosis genes. One was located adjacent to the *phe-*tRNA gene in B4-1-3 (ICE*M*sp^B4-1-3^-1) and another adjacent to the *met*-tRNA gene in CA8 (ICE*M*sp^CA8^). Both sites are documented integration sites for ICESyms. The third ICE, ICE*M*sp^B4-1-4^, was revealed to be the first example of a tripartite ICE lacking symbiosis genes.

### A tripartite ICE lacking symbiosis genes likely shares a common ancestor with the ICESym family

ICE*M*sp^B4-1-4^ (Fig. 5) is integrated into the same three chromosomal attachment sites (*phe-*tRNA, *guaA* and *met-*tRNA) as all identified tripartite ICESyms. Despite lacking the many genes required for symbiosis, ICE*M*sp^B4-1-4^ was larger than the median length for tripartite ICESyms (~560 kb) at 626 kb. ICE*M*sp^B4-1-4^ carried two gene clusters likely involved in the broad-specificity catabolism of phosphonates [74] and a plethora of metabolic enzymes with predicted involvement in fatty acid degradation, metabolism of butanoate, aspartate, asparagine, glutamate and proline.

Like other ICEs in the genus *Mesorhizobium *, ICE*M*sp^B4-1-4^ carries homologues of *rdfS* (required for ICE excision) and *fseA*, which encodes the transcriptional activator of *rdfS*, FseA. Like other ICESyms, ICE*M*sp^B4-1-4^ encodes *luxRI*-family quorum-sensing genes directly upstream of the *fseA* gene. However, the ICE*M*sp^B4-1-4^ *luxRI-*family genes are not orthologous with those on ICESyms (*traR1/traR2* and *traI1/traI2*). Comparisons revealed the ICE*M*sp^B4-1-4^ LuxR homologue shared only 30 % amino-acid identity with TraR of ICE*Ml*Sym^R7A^. ICE*M*sp^B4-1-4^ also lacked a conserved *tra*-box sequence upstream of its *luxI* homologue. Thus, ICE*M*sp^B4-1-4^ carries distinct quorum-sensing genes that nonetheless presumably control expression of *fseA* and stimulate ICE excision and transfer. ICE*M*sp^B4-1-4^ also lacks a gene for QseM, an antiactivator that binds and inhibits TraR and FseA [49]. In summary, while ICE*M*sp^B4-1-4^ closely resembles ICESyms, it lacks key regulatory genes common to all other ICESyms.

In previous work, we defined a set of 66 genes conserved on ICESyms [13] but generally absent on all other *Mesorhizobium* ICEs. Of these genes, ICE*M*sp^B4-1-4^ carries the *nadABC* for nicotinate biosynthesis, and *bioBFDAZ* for biotin biosynthesis, together present as a single cluster and sharing synteny with genes on ICE*Ml*Sym^R7A^, suggesting these vitamin synthesis genes are an ancestral feature of these ICEs. Interestingly, 115 of the NS-meso genomes lacked both *nadABC* and *bioBFDAZ* clusters, and only three NS-meso carried both operons. The only other gene present on ICE*M*sp^B4-1-4^ and conserved on ICESyms was *msi287*, which on ICE*Ml*Sym^R7A^ is present downstream of *nifH* and the nitrogenase genes. Msi287 domain composition and tertiary structure predictions suggest it is a PhoB/FixJ/MtrA-like DNA-binding response regulator. ICE*M*sp^B4-1-4^ encodes three copies of Msi287. Each copy shares 50–61 % pairwise amino-acid identity with the ICE*Ml*Sym^R7A^ copy, suggesting Msi287 paralogues are common components of several distinct regulatory systems on ICE*M*sp^B4-1-4^.

**Fig. 6.**
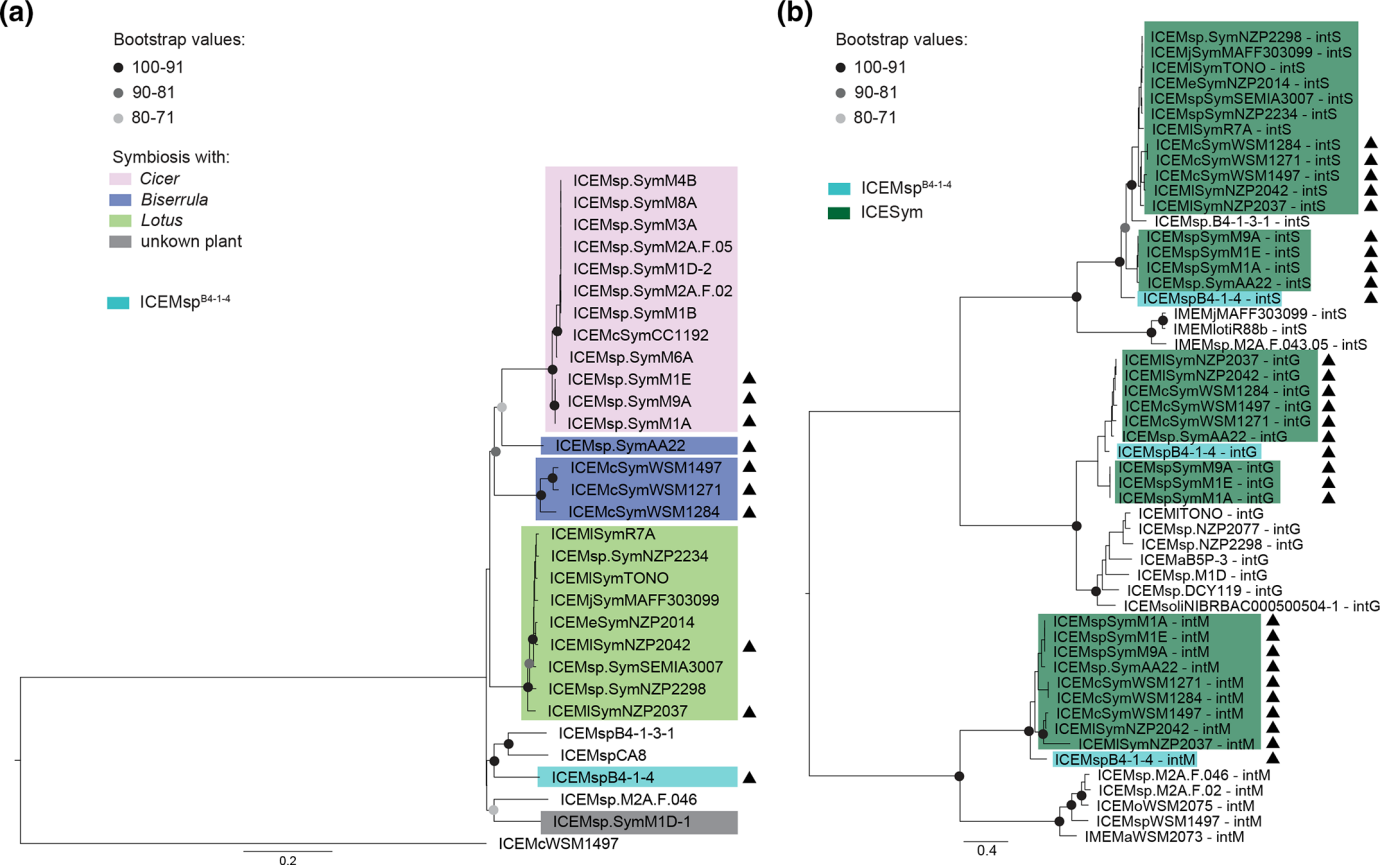
ICE*M*sp^B4-1-4^ shares a common ancestor with the archetypal ICESym. (a) Maximum-likelihood tree of ICESym clade ICEs. The tree was constructed with RAxML and it is based on the concatenation of alignments of 16 single-copy backbone genes, the tree was rooted with ICE*Mc* ^WSM1497^, scale bar indicates substitutions per site. Light green indicates the ICESym specifies symbiosis with *Lotus*, blue with *Biserrula*, pink with *Cicer*, grey with an unknown plant. The tripartite ICE*M*sp^B4-1-4^ is highlighted in azure. Triangles indicate tripartite ICEs. (b) Maximum-likelihood tree of integrases specifying integration in *guaA* (*intG*), *met*-tRNA (*intM*) and *phe*-tRNA (*intS*). The tree was constructed with RAxML and rooted at midpoint, the scale bar indicates substitutions per site. Dark green highlight indicates integrases of ICESyms, and triangles indicate the integrase belongs to a tripartite ICE. The integrases of the tripartite ICE*M*sp^B4-1-4^ are highlighted in azure.

A tree constructed from the ICE*M*sp^B4-1-4^ site-specific recombinase *intS*, *intM* and *intG,* together with related sequences on other ICEs and ICESyms [13], grouped the ICEMsp^B4-1-4^ sequences with those present on tripartite ICESyms, rather than with genes present on other ICEs that target the same integration sites (Fig. 6b). In both this tree and the tree constructed from *msi031-trb-msi021-*gene clusters (Fig. 6), ICE*M*sp^B4-1-4^ sequences were positioned at the base of the ICESym clade, suggesting ICE*M*sp^B4-1-4^ is related to an early common ancestor of tripartite ICESyms. Altogether these observations suggest the ancestor of ICEMsp^B4-1-4^ diverged from the ancestor of ICESyms early in evolution and perhaps prior to acquisition of symbiosis genes, rather than it being a relative of an extant tripartite ICESym that has subsequently lost symbiosis genes.

## Discussion

The aim of this work was to assess the indigenous *Mesorhizobium* populations in Australian soils by directly isolating them from soil and comparing their genomes with other mesorhizobia. We discovered genetically diverse indigenous *Mesorhizobium* spp. are present in Australian soil and that they overwhelmingly lack genes for nitrogen-fixing symbiosis. Isolated NS-meso shared >86 % cpAAI in pairwise comparisons with symbiotic *Mesorhizobium* spp. isolated worldwide. In core-gene trees, the NS-meso phylogeny overlapped with symbiotic strains and extended the genus by 15 genospecies. Despite lacking genes for symbiosis, NS-meso exhibited similar core genomes to symbiotic strains and harboured similar families of mobile genetic elements. These included the first discovered tripartite ICE lacking symbiosis genes, which may resemble the ancestor of extant ICESyms.

A wide diversity of nonsymbiotic *Mesorhizobium* spp. were isolated from soil, and a subset of these strains were capable of evolving into symbionts through ICESym acquisition. Although this study only focused on a limited number of sampling sites, the consistency with which NS-meso were isolated suggests that NS-meso are likely to be widespread in Australian soils. In contrast, there have been very few reports of indigenous symbiotic *Mesorhizobium* spp. isolated from Australian soils, with *Bradyrhizobium* most frequently identified in isolations from nodules of Australian native legumes [75–78]. In this study, the only symbiotic strains isolated were previously introduced inoculants or were strains that acquired ICESyms from introduced inoculants. Thus, it seems likely that the frequent isolation of novel *Mesorhizobium* microsymbionts from nodules of inoculated agricultural legumes [16, 27, 68, 79] can largely be explained by nonsymbiotic *Mesorhizobium* soil saprophytes acquiring ICESyms from inoculant strains, and not from the evolution of existing indigenous symbiotic *Mesorhizobium* spp.

The phylogenetic diversity of NS-meso isolated here overlaps the diversity of symbiotic *Mesorhizobium* strains isolated internationally. It is possible that all *Mesorhizobium* evolved from a symbiotic ancestor and in Australian soils all the extant clades subsequently lost their symbiosis ICEs (or plasmids). However, a simpler explanation is that the extant diversity observed in both nonsymbiotic and symbiotic members of the genus *Mesorhizobium* evolved prior to the arrival of genes for nitrogen-fixing symbiosis. Further genomic analysis of indigenous rhizobia isolated both from Australian native legumes and soil may help to answer this question.

Transfer of symbiosis genes between distantly related bacteria does not frequently result in an effective nitrogen-fixing symbiosis. For example, *Agrobacterium tumefaciens* carrying the symbiosis-plasmid from *R. leguminosarum* bv. *viciae* VF39 nodulates *Pisum sativum* but does not fix nitrogen [80]. Transfer of the symbiosis-plasmid pRalta from the *Mimosa-*nodulating symbiont *Cupriavidus taiwanensis* to the plant pathogen *Ralstonia solanacearum* generates a non-nodulating *Ralstonia* that eventually forms nodules following spontaneous mutations but does not fix nitrogen [81]. Thus, there are clearly barriers preventing the evolution of symbiosis through gene acquisition. These barriers likely arise from differences in the genomic backgrounds of recipients and the specific adaptation of mobile elements to those backgrounds. ICESyms are probably specifically adapted to enabling a functional symbiosis in combination with the typical core-gene complement present in members of the genus *Mesorhizobium *. Analysis of the *Mesorhizobium* core genome revealed most non-ICESym encoded genes that are likely essential for symbiosis are core genes in NS-meso. Nevertheless, as discussed above, the *Mesorhizobium* core gene complement has likely evolved independent of any involvement in nitrogen-fixing symbiosis [82]. In this work, transfer of ICEsyms *in vivo* and *in vitro* to NS-meso produced both nitrogen-fixing and non-nitrogen-fixing symbionts, consistent with observations previously reported from laboratory and field-isolated strains [17, 27]. The wide variation in symbiotic effectiveness of ICESym exconjugants and the sporadic appearance of non-fixing exconjugants likely reflects the evolutionary independence of NS-meso prior to ICESym acquisition.

Previously it was unclear if the distinct regulatory and structural features of ICESyms and tripartite ICESyms were related to their involvement in symbiosis, or if perhaps the ICESym ancestor existed in its present form prior to acquiring symbiosis genes. The tripartite ICE*M*sp^B4-1-4^ largely resembles other tripartite ICESyms but lacks genes for symbiosis. It is possible that ICE*M*sp^B4-1-4^ once was an ICESym and subsequently lost its symbiosis genes, but other differences in ICE*M*sp^B4-1-4^ suggest otherwise. The conjugation-gene sequences and site-specific recombinase sequences group basally with those of other ICESyms suggesting it branched off from them early on during its evolution. Overall, our analyses suggest ICE*M*sp^B4-1-4^ is related to an ancestor of the ICESyms that probably lacked symbiosis genes. The only genes conserved on ICE*M*sp^B4-1-4^ and all other ICESyms (aside from *msi287*) are genes for biosynthesis of nicotinic acid and biotin, which are not essential for symbiosis [83]. Interestingly, almost all of the NS-meso lacked these genes and those tested were auxotrophic for these vitamins, as were most nonsymbiotic isolates described by Sullivan *et al.* [29]. These observations suggest such vitamin auxotrophy could be advantageous for NS-meso. The lack of the *nad* genes would presumably reduce production of reactive oxygen species that contribute to bacterial death through ageing [84, 85], perhaps assisting their long-term survival in soil conditions not conducive for growth. Conversely, the vitamin prototrophy endowed by ICESyms and ICE*M*sp^B4-1-4^ might make strains more competitive in the rhizosphere during more favourable conditions.

Overall, in this work we have exposed the unrecognized diversity of the nonsymbiotic members of the *Mesorhizobium* genus. While we only interrogated a relatively small number of sites and individual organisms, it is already clear the genome diversity in the NS-meso is expansive. We suspect that the presently characterized symbiotic members of the *Mesorhizobium* represent only a very small fraction of the underlying diversity in the genus. The observation that NS-meso isolated here can form an effective symbiosis following ICESym acquisition suggests there is much potential for generation of novel symbionts with improved symbiotic effectiveness or adaptations to local soil conditions. Finally, the large genome capacity of *Mesorhizobium* genomes and the facile transfer of 100’s of kilobases of DNA via conjugation make the genus an attractive platform for rhizosphere engineering.

## Supplementary Data

Supplementary material 1

Supplementary material 2

Supplementary material 3
