# Supplementary material for: Population genomics of Australian indigenous Mesorhizobium reveals diverse nonsymbiotic genospecies capable of nitrogen-fixing symbioses following horizontal gene transfer
Source: Microb Genom. 2023 Jan 5;9(1):mgen000918. doi: 10.1099/mgen.0.000918 (PMC9973854; doi:10.1099/mgen.0.000918)
Supplement: Supplementary material 1 [file mgen-9-918-s001.pdf]

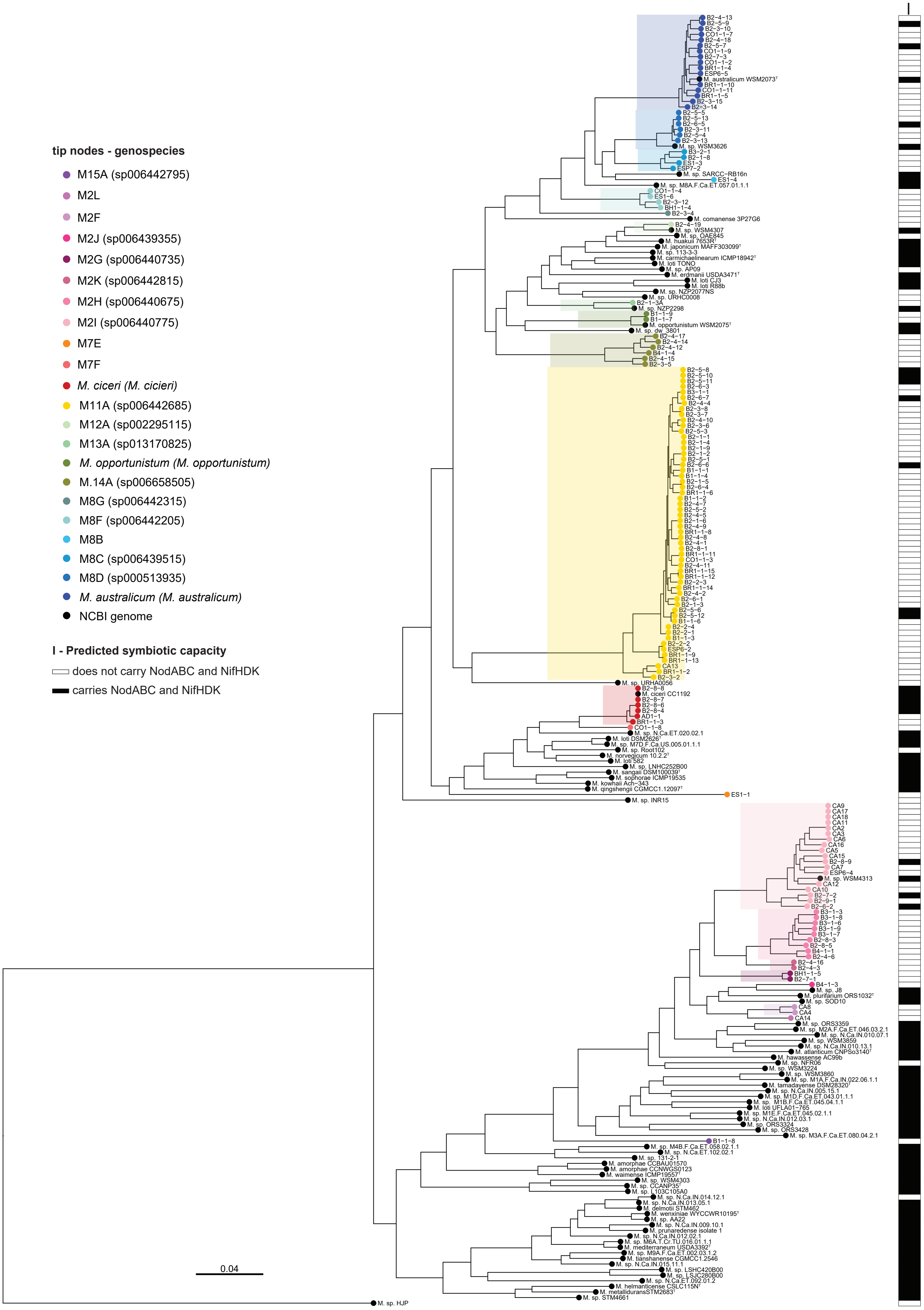

Figure S1. Core-genome phylogeny of the soil isolates and reference NCBI genomes. 1,067 single-copy core-genes were used to build maximum likelihood tree with RAxML. The tree was rooted with the M. sp. HJP. Coloured tip nodes indicate the strains were isolated in this study, with colour of the tip nodes representing the species the strain belongs to. Other genomes were downloaded from NCBI (highlighted with black tip nodes). Strains were predicted to be symbiotic if they harboured NodABC and NifHDK genes and/or if they had published symbiotic capacity, and were assumed nonsymbiotic if they lacked these genes or demonstrated symbiotic capacity. Scale bar indicates substitutions per site.

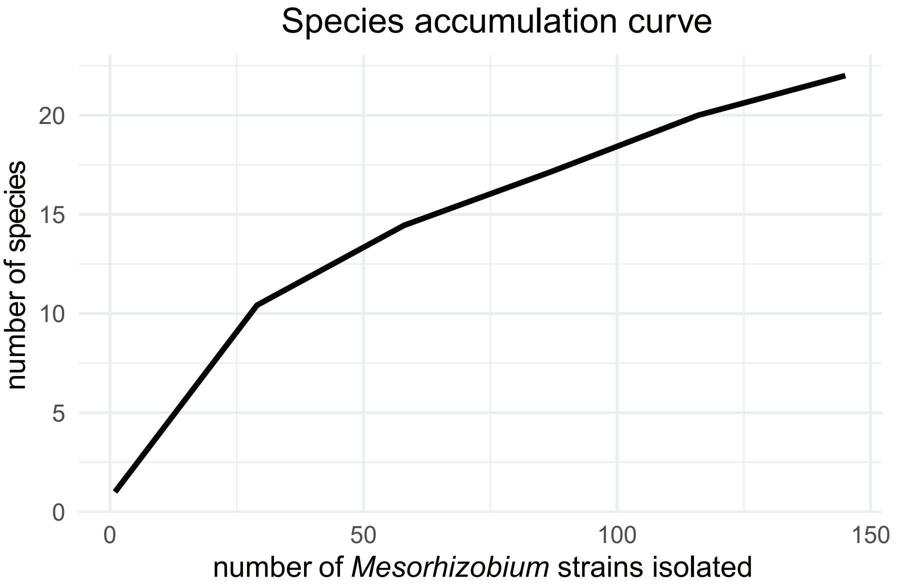

Figure S2 - Rarefaction curve. Plot of the number of species (calculated with ANI>95%) against the number of samples. The curve was calculated with the function rarefaction.single() in MOTHUR.

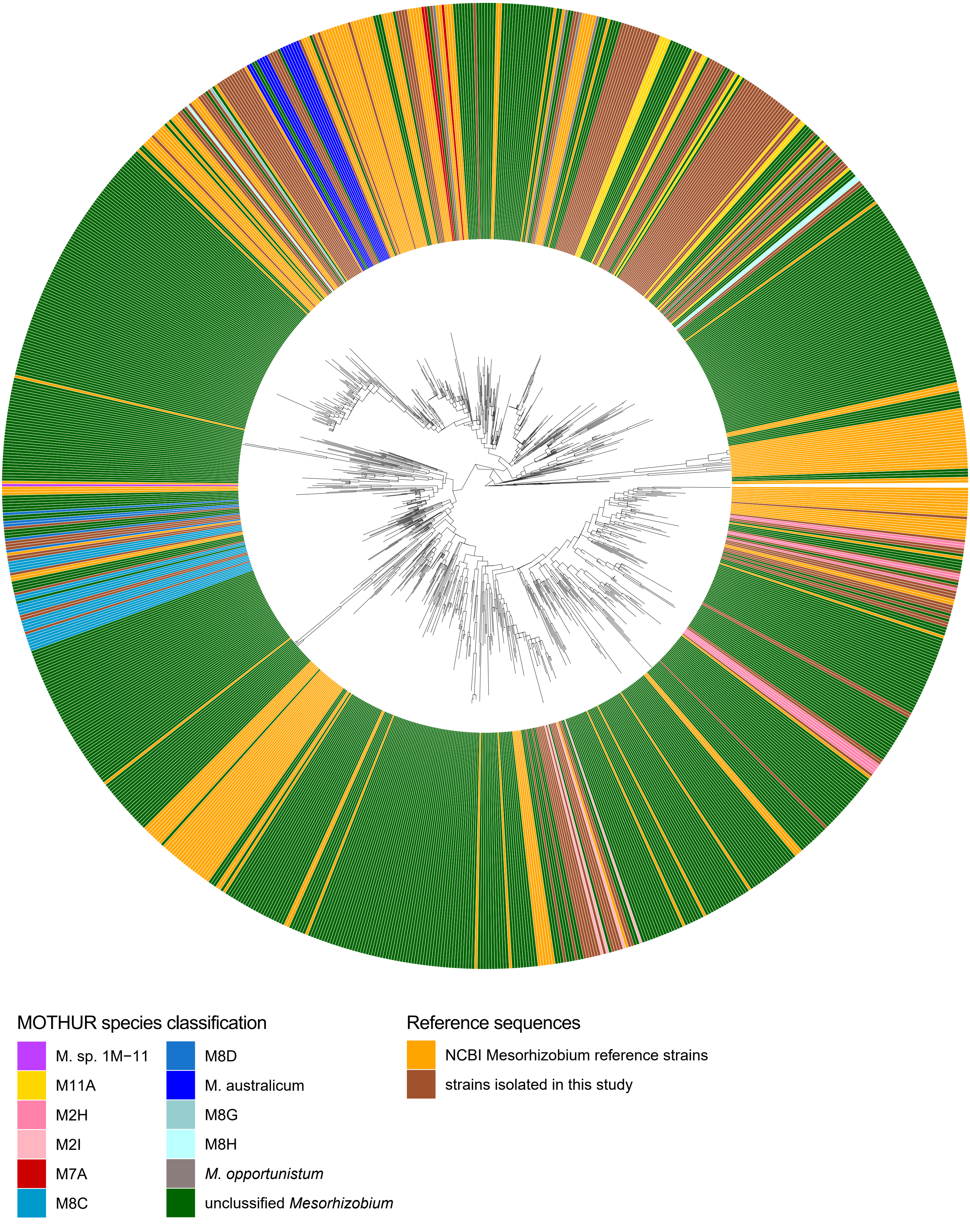

Figure S3– Phylogenetic tree of the *atpD* reads classified as *Mesorhizobium*. The reads from the microbiome analysis were aligned to *atpD* sequences of the sample strains and mesorhizobial genomes downloaded from NCBI. The external ring represents the classification of each read

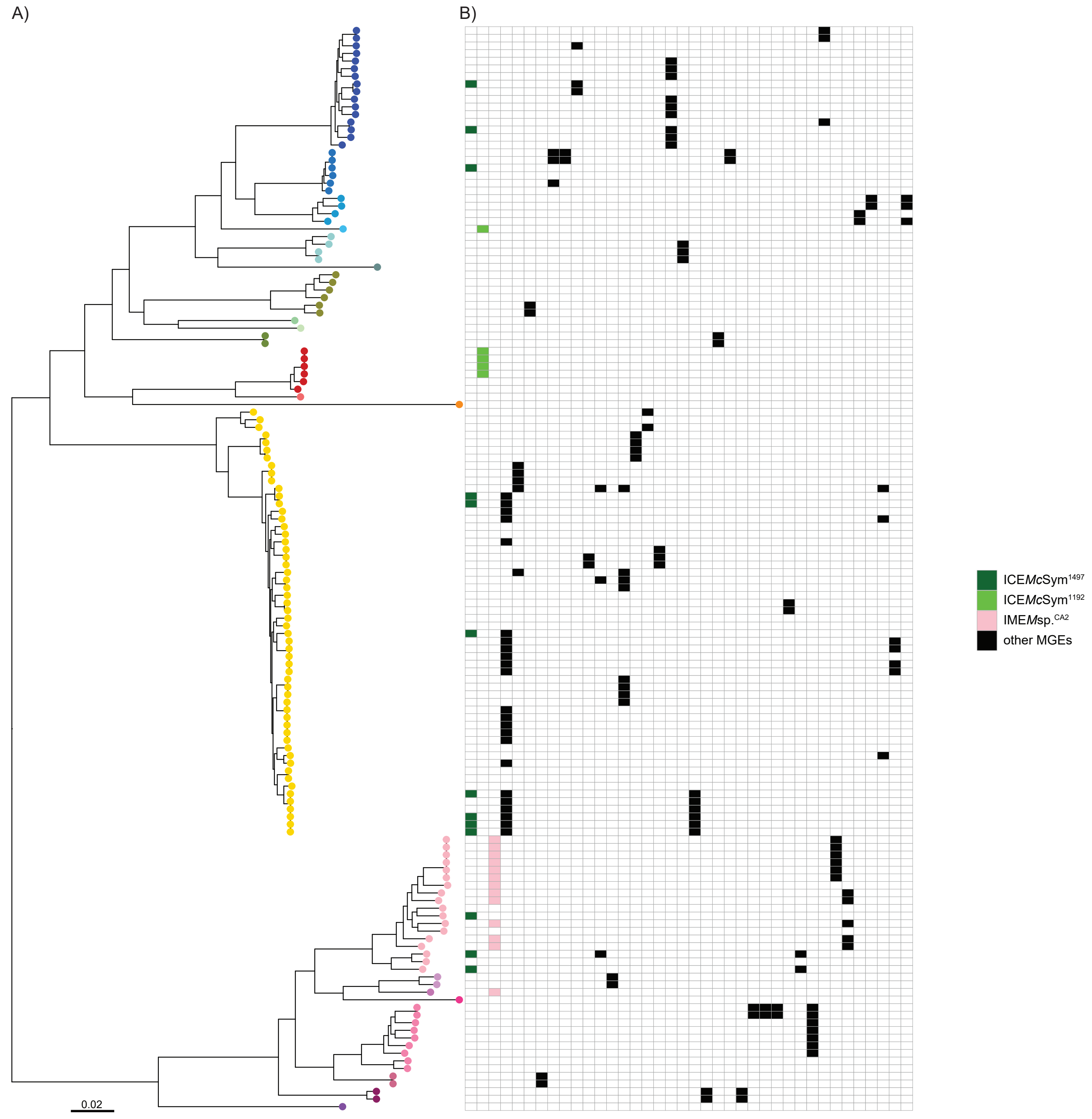

Figure S4 – Movement of MGEs across the soil-isolated strains. A) Maximum likelihood tree based on 1,067 single-copy core-genes built with RAxML. Tip nodes are coloured by genospecies with the colour-scheme of Figure 3. The tree was rooted at midpoint and the scale bar indicates substitutions per site. B) Presence and absence (in white) of MGEs. Each column represents one MGE. Each MGE was searched with BLASTn, and only MGEs present in more than one strains were displayed.

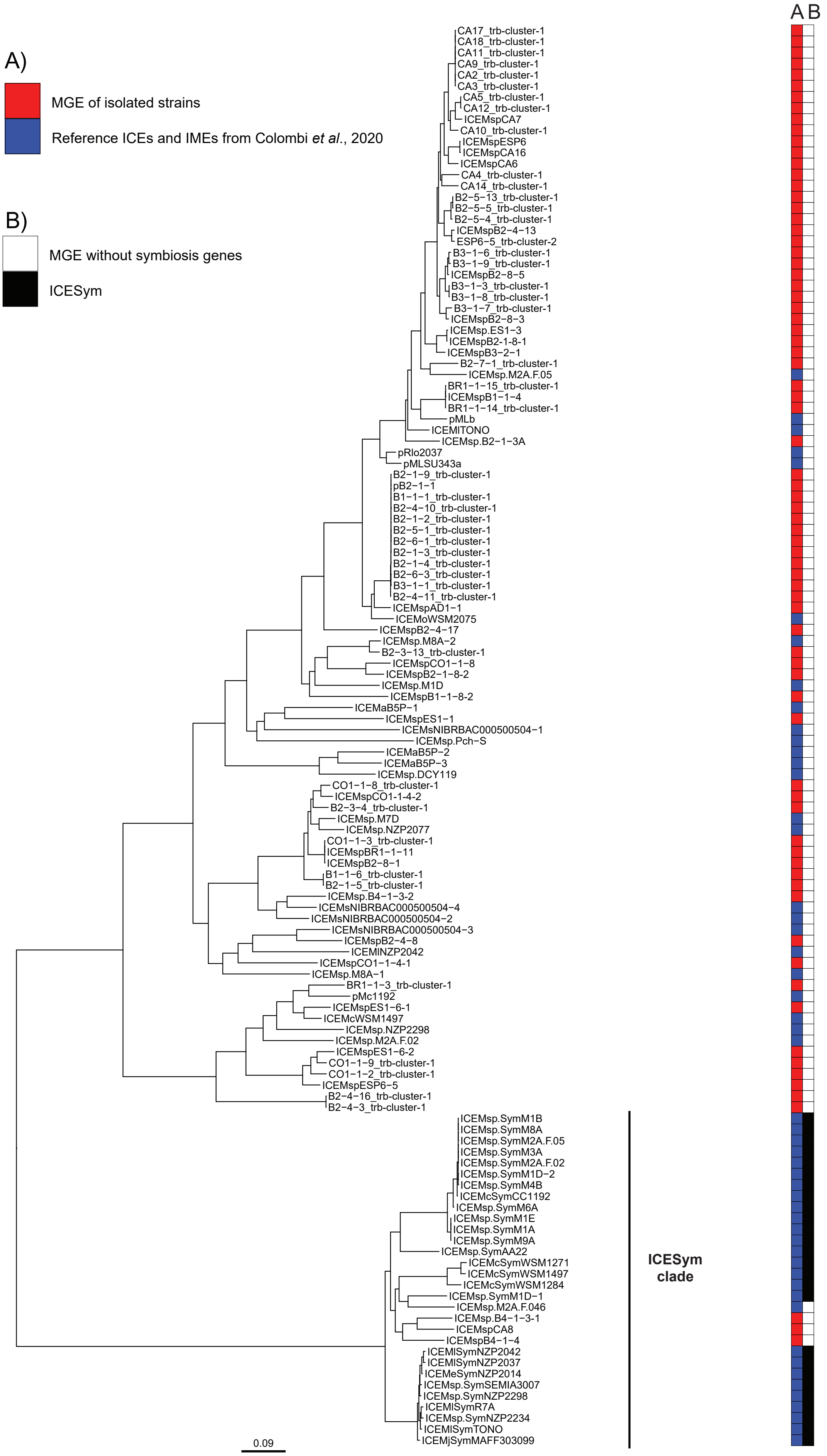

Figure S5 - Maximum likelihho tree of the trb clusters. The RAxML tree was rooted at midpoint and the scale bar indicates substitutions per site. A) indicates if the MGE was present in the soil-isolated strain or was a MGE described in Colombi et al., 2020; B) indicates if the element is an ICESym.
